# Increased IL‐23 is Associated to Heliotrope Sign in Multivariate Model on Idiopathic Inflammatory Myopathies

**DOI:** 10.1155/drp/4660804

**Published:** 2026-10-21

**Authors:** Christian Juarez-Gomez, Oscar Pizano-Martinez, María Guadalupe Ramírez-Dueñas, Cynthia-Alejandra Gomez-Rios, Maribell Cabrera-López, Arcelia Llamas-García, Andrea Aguilar-Vazquez, Monica Vazquez-Del-Mercado

**Affiliations:** ^1^ Instituto de Investigación en Reumatología y del Sistema Músculo-Esquelético (IIRSME) Departamento de Biología Molecular y Genómica Centro Universitario de Ciencias de la Salud Universidad de Guadalajara Guadalajara Jalisco Mexico udg.mx; ^2^ Departamento de Fisiología Centro Universitario de Ciencias de la Salud Universidad de Guadalajara Guadalajara Jalisco Mexico udg.mx; ^3^ División de Medicina Interna Servicio de Reumatología SNP 004086-SECIHTI Nuevo Hospital Civil “Dr. Juan I. Menchaca” Guadalajara Jalisco Mexico

## Abstract

Heliotrope rash is a pathognomonic feature of dermatomyositis (DM); however, the molecular pathways implicated in cutaneous involvement have not been fully explored. Although the IL‐23/Th17 axis has been implicated in inflammatory and autoimmune diseases, the clinical significance of circulating IL‐23 across the idiopathic inflammatory myopathy (IIM) spectrum, particularly in cutaneous disease expression, remains insufficiently defined. In this cross‐sectional study, we aimed to quantify serum IL‐23 levels in patients with IIM and evaluate their association with cutaneous involvement, including overall skin disease activity assessed by the Cutaneous Dermatomyositis Disease Area and Severity Index (CDASI) and the presence of specific skin manifestations, particularly pathognomonic lesions, using a univariate analysis. Serum IL‐23 levels were measured by Enzyme‐Linked Immunosorbent Assay (ELISA) in patients with IIM and healthy controls (HCs), and their relationships with clinical and cutaneous variables were analyzed using univariable and multivariable approaches. Higher IL‐23 levels were associated with cutaneous involvement, especially with pathognomonic skin manifestations and, most notably, heliotrope sign. IL‐23 also showed a positive correlation with CDASI activity; the relationship with heliotrope sign remained consistent. Applying a multivariate model, heliotrope rash remained the only independent factor associated with IL‐23 levels: in other words, IL‐23 increases by 437.3 pg/mL when heliotrope is present. These findings suggest that IL‐23 may be preferentially associated with a cutaneous phenotype characterized by heliotrope rash, rather than with global skin burden alone.

## 1. Introduction

Despite the well‐recognized importance of IL‐23 in cutaneous involvement in juvenile dermatomyositis (DM), in contrast, its role in adult DM has not been similarly explored. Most studies in DM have focused on muscle weakness, systemic organ involvement, autoantibody profiles, or overall disease activity. In general, the assessment of core set measures, such as physician global assessment (PGA), patient global assessment (PtGA), manual muscle testing‐8 (MMT‐8), and creatine phosphokinase (CPK), has been prioritized, whereas skin‐related features have received less attention from a biomarker‐oriented perspective [1, 2].

Idiopathic inflammatory myopathy (IIM) is a heterogeneous group of autoimmune disorders characterized by skeletal muscle inflammation and variable extramuscular manifestations [3]. Among them, DM is distinguished by its prominent cutaneous involvement, which includes pathognomonic lesions such as heliotrope and Gottron’s sign [4]. Beyond their diagnostic relevance, these lesions reflect complex inflammatory processes involving both innate and adaptive immune responses [5].

Increasing evidence suggests that cytokine‐mediated pathways play a substantial role in the pathogenesis of IIM. In particular, the IL‐23/Th17 axis has emerged as a relevant immunological pathway in some autoimmune diseases, where it promotes sustained inflammation and tissue injury [6]. In DM, emerging evidence implicates this pathway in cutaneous inflammation, supporting a potential role for IL‐23 in the immunobiology of skin involvement.

In psoriasis, IL‐23 is recognized as a central driver of cutaneous inflammation [7]. Consistent with this, experimental models have shown that skin‐specific overexpression of IL‐23 can induce a psoriatic arthritis (PsA)‐like phenotype in K23 mice, underscoring the pathogenic relevance of this cytokine in skin‐driven inflammatory disease [8]. In contrast, although DM seems to share elements of the IL‐23/Th17 axis with psoriasis, the extent to which IL‐23 contributes to cutaneous manifestations in DM classical, hypomyopathic, or amyopathic remains insufficiently defined [7, 9]. Although Cutaneous Dermatomyositis Disease Area and Severity Index (CDASI) score [10] is useful for assessing overall skin disease activity and damage, it remains important to determine whether IL‐23 is associated with the global cutaneous burden or individual cutaneous manifestations, particularly pathognomonic features. Therefore, this cross‐sectional study aimed to quantify serum IL‐23 levels in patients with IIM and evaluate their association with cutaneous involvement, including cutaneous disease activity assessed by the CDASI.

## 2. Methods

### 2.1. Patients

This cross‐sectional preliminary study included 33 Mexican individuals with IIM consecutively recruited at Hospital Civil de Guadalajara “Dr. Juan I. Menchaca,” a tertiary referral center in Guadalajara, Mexico. Eligible participants were IIM patients classified according to the Bohan and Peter criteria [11, 12], the 2017 European League Against Rheumatism/American College of Rheumatology (EULAR/ACR) classification criteria [13], and the 2004, 2011, and 2023 European Neuromuscular Centre (ENMC) criteria [14–16]. Healthy controls (HCs) included 2 men and 9 women, with an age range of 22–57 years. Given the exploratory and preliminary nature of the study, demographic characteristics were considered during recruitment to maintain an overall comparable reference group.

All participants provided written informed consent before enrollment. The study was conducted in accordance with the Declaration of Helsinki 2013, and it was approved by the Ethics and Research Committees of Hospital Civil de Guadalajara “Dr. Juan I. Menchaca” (Secretaría de Salud Jalisco registration number 0657/23 HCJIM/2023). Peripheral blood was collected from each subject; serum was separated by centrifugation (3500 rpm for 10 min) and stored at −80°C until analysis.

### 2.2. Clinical Assessment

Core set measures were performed by experienced rheumatologists. Cutaneous manifestations were recorded through direct clinical examination and included pathognomonic (heliotrope rash and Gottron’s sign) and nonpathognomonic skin findings. Cutaneous disease activity was assessed using the CDASI [10], a validated instrument designed to quantify the extent and severity of DM‐related skin involvement across activity and damage domains. Briefly, the CDASI activity score giving 0–4 in Likert scale in last four weeks of the patient visit evaluates erythema, scale, and excoriation/erosion‐ulceration across multiple anatomical sites, together with additional items such as Gottron’s sign, periungual changes, and alopecia. Muscle enzyme levels were obtained from patient clinical records as part of the routine workup.

### 2.3. Determination of IL‐23 Serum Levels

Serum IL‐23 levels were quantified by sandwich Enzyme‐Linked Immunosorbent Assay (ELISA) (Human IL‐23 ELISA Kit, Invitrogen/Thermo Fisher Scientific, Waltham, MA, USA; BMS2023‐3) following the manufacturer’s instructions. Standards (15.6–2000 pg/mL) and samples were run in duplicate, and absorbance was read at 450 nm (reference 620 nm) on a Multiskan SKY microplate spectrophotometer (thermoscientific spectrophotometer, Multiskan Sky with Cuvette and Touch Screen, Made in Singapore; ref 51119700); concentrations were interpolated using a 5‐parameter logistic (5‐PL) standard curve. Serum samples from patients and HC were processed and analyzed under identical experimental conditions.

### 2.4. Statistical Analysis

Data distribution was examined with the Kolmogorov–Smirnov test. Numerical variables are summarized as mean with range (min–max), whereas categorical variables are expressed as percentages. Comparisons of continuous variables were performed using the Mann–Whitney *U* test, and associations were evaluated using Spearman’s rank correlation coefficient; categorical variables were analyzed using the chi‐square test or Fisher’s exact test, as appropriate.

IL‐23 levels and CDASI activity scores were analyzed as continuous variables in correlation and regression analyses. For subgroup comparisons, cutaneous disease activity was additionally categorized as absent (CDASI = 0) or present (CDASI > 0). Sensitivity analyses were performed restricting the cohort to DM patients to evaluate the robustness of associations between IL‐23 levels and cutaneous manifestations.

A multivariable linear regression model was fitted using the enter method, considering IL‐23 as the dependent variable. Clinically relevant variables considered potential confounders were selected a priori and included CDASI activity score, diagnosis (DM vs. non‐DM), and the presence of heliotrope rash. Multicollinearity was assessed using tolerance and variance inflation factor (VIF), with no evidence of significant collinearity observed. Model assumptions were evaluated, and no relevant autocorrelation of residuals was detected (Durbin–Watson statistic).

Missing clinical data (*n* = 1) were minimal and were handled using complete‐case analysis for the corresponding analyses. Serum IL‐23 levels were available for all participants. Statistical analyses were performed in SPSS v27.0 (SPSS Inc., Chicago, IL, USA) and GraphPad Prism v10.0 (GraphPad Software, La Jolla, CA, USA), and two‐tailed *p* values < 0.05 were considered statistically significant.

## 3. Results

### 3.1. Clinical Characteristics of IIM Patients

In this preliminary report, 33 patients with IIM fulfilling the 2017 EULAR/ACR [13], Bohan and Peter [11, 12], and 2004, 2011, and 2023 ENMC [14–16] classification criteria were included (seven men, 21.2%; 26 women, 78.8%). For comparative purposes, we classified the patients into two groups: DM (*n* = 20) and non‐DM (*n* = 13), the latter including polymyositis (PM, *n* = 3), inclusion body myositis (IBM, *n* = 1), immune‐mediated necrotizing myopathy (IMNM, *n* = 1), cancer‐associated myositis (CAM, *n* = 3), antisynthetase syndrome (ASyS, *n* = 1), and overlap myositis (OV, *n* = 4), given the limited sample size across individual subtypes. In addition, 11 HCs were included. Demographic, clinical, and biochemical characteristics are summarized in Table 1. No patients had documented UV light exposure. None presented with mechanic’s hands, capillaroscopic abnormalities, ulceration, gastrointestinal perforation, abdominal pain, vasculitis, or arrhythmias. Anti‐Jo1, Anti‐PL7, Anti‐PL12, and Anti‐OJ antibodies were not detected in any patient.

**TABLE 1:** Demographic, clinical, and biochemical data of IIM patients.

Feature	DM group (*n* = 20)	Non‐DM group (*n* = 13)	*p* value
Demographic characteristics			
Age [years, $\underset{\_}{x}$ (min–max)]	47.10 (12.34–77.13)	52.45 (31.26–73.69)	0.632^**$**^
Clinical characteristics			
Disease duration diagnosis [years, $\underset{\_}{x}$ (min–max)]	5.07 (0.32–27.52)	4.34 (0.00–14.17)	0.939^**$**^
Disease duration symptoms [years, $\underset{\_}{x}$ (min–max)]	4.58 (0.00–27.52)	3.46 (0.00–14.00)	0.983^**$**^
MMT‐8 [score, $\underset{\_}{x}$ (min–max)]	144.45 (110.00–150.00)	144.18 (128.00–150.00)	0.458^**$**^
MYOACT [score, $\underset{\_}{x}$ (min–max)]	0.04 (0.00–0.27)	0.05 (0.00–0.18)	0.395^**$**^
MDI extent [score, $\underset{\_}{x}$ (min–max)]	0.02 (0.00–0.08)	0.03 (0.00–0.11)	0.629^**$**^
PtGA [score, $\underset{\_}{x}$ (min–max)]	20.00 (0.00–60.00)	41.67 (0.00–90.00)	0.390^**$**^
PGA [score, $\underset{\_}{x}$ (min–max)]	18.00 (0.00–40.00)	28.33 (0.00–90.00)	0.972^**$**^
CDASI activity [score, $\underset{\_}{x}$ (min–max)]	3.00 (0.00–8.00)	0.15 (0.00–1.00)	**0.003** ^**$**^
Laboratory parameters			
CRP [U/L, $\underset{\_}{x}$ (min–max)]	4.42 (0.10–10.00)	13.04 (0.50–44.87)	0.573^**$**^
ESR [U/L, $\underset{\_}{x}$ (min–max)]	28.33 (1.00–62.00)	22.14 (3.00–35.00)	0.821^**$**^
CPK [U/L, $\underset{\_}{x}$ (min–max)]	257.29 (24.00–1746.00)	1616.11 (27.00–6190.00)	0.711^**$**^
Aldolase [U/L, $\underset{\_}{x}$ (min–max)]	3.32 (1.20–7.90)	5.70 (1.10–8.10)	0.476^**$**^
AST [U/L, $\underset{\_}{x}$ (min–max)]	39.05 (11.70–228.00)	68.20 (12.10–236.00)	0.311^**$**^
ALT [U/L, $\underset{\_}{x}$ (min–max)]	28.45 (13.00–90.00)	105.40 (13.00–430.00)	0.604^**$**^
LDH [U/L, $\underset{\_}{x}$ (min–max)]	398.62 (96.00–1872.00)	425.80 (160.00–1009.00)	0.707^**$**^
Personal History			
Hypertension *n* (%)	3 (15.0)	1 (7.7)	0.641^**¢**^
Diabetes *n* (%)	—	2 (15.4)	0.142^**¢**^
Alcohol *n* (%)	1 (5.0)	1 (7.7)	> 0.999^**¢**^
Smoke *n* (%)	3 (15.0)	1 (7.7)	0.641^**¢**^
Drugs *n* (%)	1 (5.0)	1 (7.7)	> 0.999^**¢**^
Extramuscular manifestations			
Arthritis *n* (%)	3 (15.0)	3 (23.1)	0.647^**¢**^
Arthralgia *n* (%)	3 (15.0)	2 (15.4)	> 0.999^**¢**^
Joint contracture *n* (%)	1 (5.0)	—	> 0.999^**¢**^
Raynaud’s phenomenon *n* (%)	—	1 (7.7)	0.375^**¢**^
Heliotrope *n* (%)	9 (45.0)	—	**0.012** ^**¢**^
Gottron’s sign *n* (%)	8 (40.0)	—	**0.030** ^**¢**^
Shawl sign *n* (%)	3 (15.0)	—	0.274^**¢**^
V sign *n* (%)	8 (40.0)	—	**0.030** ^**¢**^
Periungual erythema *n* (%)	1 (5.0)	—	> 0.999^**¢**^
Dysphagia *n* (%)	2 (10.0)	2 (15.4)	> 0.999^**¢**^
Calcinosis *n* (%)	1 (5.0)	1 (7.7)	> 0.999^**¢**^
Alopecia *n* (%)	1 (5.0)	2 (15.4)	> 0.999^**¢**^
Interstitial lung disease *n* (%)	3 (15.0)	2 (15.4)	> 0.999^**¢**^
Pulmonary hypertension *n* (%)	—	1 (7.7)	0.375^**¢**^
Cardiomyopathy *n* (%)	1 (5.0)	1 (7.7)	> 0.999^**¢**^
Treatment			
Methotrexate *n* (%)	11 (55.0)	8 (61.5)	0.466^**¢**^
Prednisone *n* (%)	14 (70.0)	7 (53.8)	0.687^**¢**^
Hydroxychloroquine *n* (%)	8 (40.0)	4 (30.8)	> 0.999^**¢**^
Mycophenolate mofetil *n* (%)	5 (25.0)	4 (30.8)	0.687^**¢**^
Azathioprine *n* (%)	3 (15.0)	1 (7.7)	> 0.999^**¢**^
Autoantibodies presence			
Anti‐Mi2	3 (15.0)	1 (7.7)	0.638^**¢**^
Anti‐Mi2 *α*	3 (15.0)	1 (7.7)	0.638^**¢**^
Anti‐Mi2 *β*	3 (15.0)	—	0.261^**¢**^
Anti‐TIF1 *γ*	4 (20.0)	—	0.136^**¢**^
Anti‐MDA5	2 (10.0)	3 (23.1)	0.360^**¢**^
Anti‐NXP2	1 (5.0)	—	> 0.999^**¢**^
Anti‐SAE	5 (25.0)	—	0.131^**¢**^
Anti‐SRP	—	1 (7.7)	0.394^**¢**^
Anti‐cN1A	—	1 (7.7)	0.200^**¢**^
Anti‐Ku	—	1 (7.7)	0.394^**¢**^
Anti‐PMScl100	—	2 (15.4)	0.148^**¢**^
Anti‐PMScl75	—	1 (7.7)	0.394^**¢**^
Anti‐Ro52	6 (30.0)	4 (30.8)	> 0.999^**¢**^
Anti‐EJ	—	2 (15.4)	0.148^**¢**^

*Note:* Data are presented as mean (min–max) or *n* (%), as appropriate. Continuous variables were compared using the Mann–Whitney *U* test (^**$**^), and categorical variables using the chi‐square test (^**¢**^).ALT: alanine aminotransferase; AST: aspartate aminotransferase; CPK: creatine phosphokinase; DM: dermatomyositis; MYOACT: Myositis Disease Activity Assessment Tool; LDH: lactate dehydrogenase; PtGA: Patient Global Assessment; CDASI, Cutaneous Dermatomyositis Disease Area and Severity Index. Bold indicates statistically significant values.

Abbreviations: CRP, C‐reactive protein; ESR, erythrocyte sedimentation rate; MDI, Myositis Damage Index; MMT8, manual muscle testing 8; PGA, Physician Global Assessment.

### 3.2. IL‐23 Serum Levels are Increased in IIM and Associated With Cutaneous Involvement

IL‐23 levels were markedly increased in patients with IIM compared with HC (Figure 1A, *p* < 0.001). Although the CDASI was originally developed for the assessment of cutaneous activity in DM, we applied it across the entire IIM cohort as an exploratory approach to examine whether cutaneous disease burden captured by this instrument was aligned with IL‐23 levels beyond the DM phenotype. In this context, patients with a CDASI activity score > 0 exhibited significantly higher IL‐23 levels than those with a CDASI activity score = 0 (Figure 1B, *p* = 0.031), and IL‐23 levels showed a positive correlation with CDASI activity score (Figure 1C, *r*_*s*_ = 0.406, *p* = 0.019). As expected, CDASI activity scores were significantly higher in patients with DM than in non‐DM IIM subgroups (Figure 1D, *p* = 0.001), supporting the biological and clinical coherence of this analysis. Consistent with these findings, IL‐23 levels were higher in patients with overall cutaneous manifestations than in those without skin involvement (Figure 1E, *p* = 0.048) and were also significantly increased in patients with pathognomonic cutaneous manifestations compared with those without such features (Figure 1F, *p* = 0.017). Likewise, patients presenting heliotrope rash exhibited higher IL‐23 levels than those lacking this manifestation (Figure 1G, *p* = 0.002).

**FIGURE 1:**
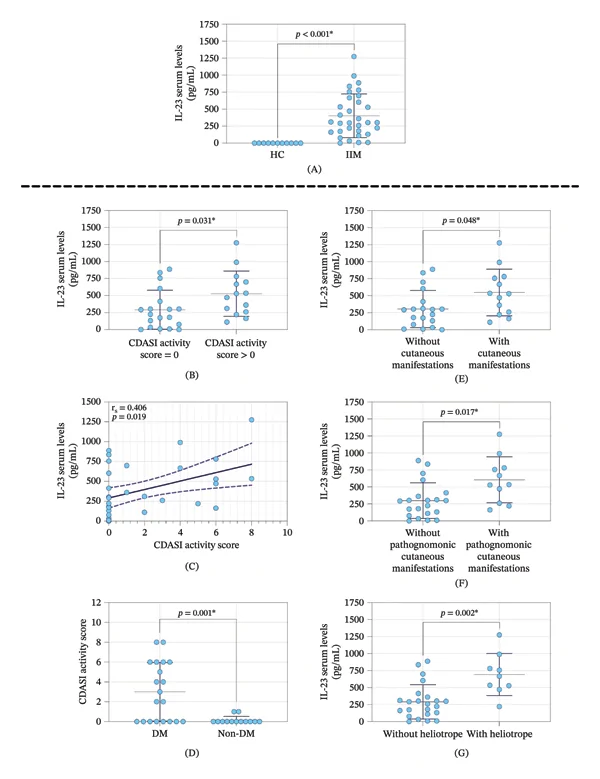
Association of serum IL‐23 levels with CDASI and cutaneous involvement in IIM. (A) Comparison of IL‐23 levels between HC and patients with IIM. (B) IL‐23 levels according to CDASI activity score (0 vs. > 0). (C) Correlation between IL‐23 levels and CDASI activity score. (D) Comparison of CDASI activity scores between DM and no DM patients. (E) IL‐23 levels according to the presence of pathognomonic cutaneous manifestations. (F) IL‐23 levels according to the presence of heliotrope. (G) IL‐23 levels according to the presence of cutaneous manifestations. HC: healthy controls; IIMs: idiopathic inflammatory myopathies; CDASI: Cutaneous Dermatomyositis Disease Area and Severity Index; DM: dermatomyositis; ^∗^Mann–Whitney *U* test.

Regarding treatment at the time of serum collection, no significant differences were observed between DM and non‐DM patients in the use of methotrexate, prednisone, hydroxychloroquine, mycophenolate mofetil, or azathioprine (all *p* > 0.05). None of the patients were receiving biologic therapies. Furthermore, serum IL‐23 levels did not significantly differ according to exposure to any of the treatments.

### 3.3. Association Between IL‐23 and Cutaneous Involvement Is Maintained in DM Phenotype

When the analysis was restricted to DM patients, similar patterns to those observed in the overall cohort were identified. IL‐23 levels tended to be higher in patients with a CDASI activity score > 0 than in those with a score of 0 (Table 1 and Figure 2A, *p* = 0.181), and a positive trend was also observed between IL‐23 levels and CDASI activity score (Figure 2B, *r*_*s*_ = 0.429, *p* = 0.059). Likewise, patients with DM in apparent clinical cutaneous remission tended to exhibit higher IL‐23 levels than those without skin involvement (Figure 2C, *p* = 0.069). In contrast, IL‐23 levels were significantly higher in patients with pathognomonic cutaneous manifestations, namely, Gottron’s sign and heliotrope rash, than in those without these features (Figure 2D, *p* = 0.020). Consistently, heliotrope sign analyzed as an individual feature was also associated with increased IL‐23 levels (Figure 2E, *p* = 0.002).

**FIGURE 2:**
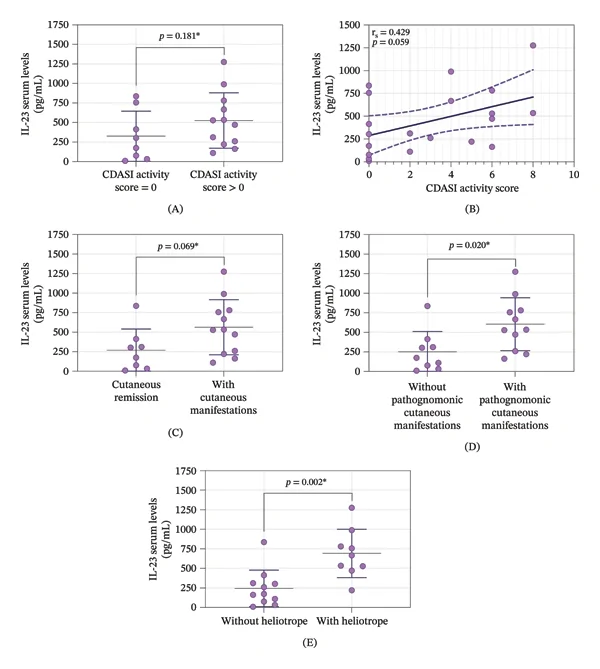
Association of serum IL‐23 levels with CDASI and cutaneous involvement in DM. (A) IL‐23 levels according to CDASI activity score (0 vs. > 0). (B) Correlation between IL‐23 levels and CDASI activity score. (C) IL‐23 levels according to the presence or absence of pathognomonic cutaneous manifestations. (D) IL‐23 levels according to the presence or absence of heliotrope rash. (E) IL‐23 levels according to the presence or absence of cutaneous manifestations. CDASI: Cutaneous Dermatomyositis Disease Area and Severity Index; DM: dermatomyositis; ^**∗**^Mann–Whitney *U* test.

### 3.4. IL‐23 Serum Levels are Associated to Heliotrope Sign in a Multivariate Model

To identify clinical factors independently associated with IL‐23, a multivariable linear regression model was performed with IL‐23 as the dependent variable. Based on our findings, CDASI activity score, diagnosis (DM vs. non‐DM), and the presence of heliotrope rash were included as independent variables.

In this model, the presence of heliotrope sign was associated with IL‐23 as a biological marker (*B* = 437.3 pg/mL, *p* = 0.017): in other words, IL‐23 increases by 437.3 pg/mL when heliotrope is present. These findings suggest that IL‐23 may be preferentially associated with a cutaneous phenotype characterized by heliotrope rash, rather than global skin burden alone. The overall model was statistically significant (*p* = 0.008) and explained approximately 27% of the variability in IL‐23 levels (adjusted *R* ^2^ = 0.268) (Table 2).

**TABLE 2:** Clinical factors associated with IL‐23 serum levels in multivariable regression analysis.

Variable	B (pg/mL)	95% CI	Standardized β	*p* value
Diagnosis (DM or non‐DM)	89.9	−153 to 333	0.14	0.46
CDASI activity (score)	2.38	−58 to 63	0.02	0.94
Heliotrope rash (presence or absence)	437.3	85 to 789	0.62	**0.017**

*Note:* B values represent unstandardized regression coefficients, and β values represent standardized coefficients. No evidence of autocorrelation was detected (Durbin–Watson = 1.34). No multicollinearity was observed among covariates (all VIF < 3). DM, dermatomyositis; CDASI, Cutaneous Dermatomyositis Disease Area and Severity Index. Bold indicates the statistically significant value.

Abbreviation: CI, confidence interval.

## 4. Discussion

In this study, higher serum IL‐23 levels were associated with cutaneous involvement, specifically with pathognomonic skin manifestations and, most notably, with heliotrope rash. In parallel, IL‐23 demonstrated a positive correlation with CDASI activity, supporting a link between this cytokine and the cutaneous involvement. These findings are relevant because they suggest that IL‐23 could be connected to clinically recognizable skin features in DM and related to IIM.

This interpretation is biologically plausible considering the known role of IL‐23 as an upstream cytokine in the IL‐23/Th17 axis [17]. Previous studies in adult PM and DM have shown increased serum IL‐23, particularly in an early state of disease [18]. Within this framework, our results extend the existing literature by proposing that the relevance of IL‐23 lies in the cutaneous domain.

Considering that CDASI is a tool for assessing overall cutaneous disease activity in DM, this might imply that IL‐23 may be more closely associated with individual cutaneous manifestations than with global skin burden.

One possible molecular explanation is that IL‐23, acting upstream of the Th17 axis, may promote the expansion and stabilization of IL‐17‐related immune responses, which in turn can activate keratinocytes and induce the production of proinflammatory chemokines and antimicrobial peptides, thereby sustaining local skin inflammation. Evidence from psoriasis supports this type of IL‐23‐driven inflammatory circuitry, in which downstream mediators contribute to a self‐reinforcing cutaneous inflammatory loop [19]. Although this mechanism has not been directly demonstrated in DM skin, it could represent one of the molecular pathways underlying the observed relationship between IL‐23 and individual cutaneous manifestations.

The multivariable model provided the clearest support for this interpretation. After adjustment, heliotrope rash remained the only independent factor associated with IL‐23 levels, whereas CDASI activity and the diagnosis of DM were not significant. This finding provides the most refined interpretation of the observed associations. It suggests that the relationship between IL‐23 and CDASI may be driven, at least in part, by the contribution of heliotrope rash within the broader skin activity profile.

The fact that DM itself did not remain independently associated with IL‐23 in the adjusted model is also important. This finding argues against the skin clinical activity on the patient recruitment with DM and instead supports the concept of biological heterogeneity within the disease. DM is increasingly understood as a diverse condition in which distinct clinical organ involvement manifestations may reflect partially different inflammatory pathways [20]. Our data fit this model and suggest that IL‐23 is preferentially associated with a phenotype characterized by heliotrope rash, rather than with DM.

From a mechanistic standpoint, this interpretation is consistent with prior experimental evidence. In murine models of inflammatory myositis (C protein‐induced myositis), IL‐23 has been detected in infiltrating macrophages within injured muscle, and disruption of the IL‐23 pathway attenuates disease severity [21]. Although those studies focused mainly on muscle tissue, they support the broader idea that IL‐23 may participate in tissue‐specific inflammatory programs in myositis.

This concept also aligns with experimental psoriatic disease models, including a PsA model generated by skin‐specific overexpression of IL‐23, which supports the ability of IL‐23‐driven cutaneous inflammation to extend beyond the skin and promote broader inflammatory phenotypes [8]. In parallel, clinical and epidemiologic data further suggest that psoriasis, PsA, and DM may share elements of the IL‐17/IL‐23 inflammatory axis, reinforcing the biological plausibility that IL‐23 contributes to selected cutaneous phenotypes [7].

From a clinical perspective, these results may also help explain why pathway‐level biological plausibility does not always translate into a clear therapeutic benefit. Trials targeting the IL‐12/23 axis in broad PM/DM populations have not shown clear efficacy, suggesting that this pathway may not be uniformly dominant across all patients [22].

Our study has limitations that should be acknowledged. Its cross‐sectional design precludes conclusions about causality, the single‐center cohort may limit the generalizability of the findings, and the sample size limits the precision of subgroup analyses and multivariable estimates. In addition, serum IL‐23 may not fully reflect local cytokine activity within skin lesions, and IL‐23 in situ expression was not assessed.

Most patients were receiving immunosuppressive or immunomodulatory therapy at the time of serum collection, which could potentially influence circulating cytokine levels. Although treatment distribution was similar between DM and non‐DM groups and IL‐23 levels did not significantly differ according to individual treatment exposure, a treatment‐related effect cannot be completely excluded given the limited sample size and observational design.

Furthermore, the clinical and immunopathological heterogeneity of the non‐DM group may have limited our analyses and the ability to detect differences in muscle involvement between groups. Although serum IL‐23 was not significantly associated with muscle‐associated enzymes or MMT‐8, a potential contribution of muscle involvement to circulating IL‐23 levels cannot be excluded. Nevertheless, the use of a cutaneous activity score together with lesion‐specific analyses and multivariable modeling represents an important strength, as it allowed a more refined interpretation of the IL‐23 findings. Further studies in larger cohorts are warranted to confirm and extend these findings.

Another limitation is that our findings suggest that circulating IL‐23 is preferentially associated with a heliotrope‐associated cutaneous phenotype within the IIM spectrum. However, these results do not establish disease or cytokine specificity and should be validated through broader cytokine profiling and comparison with other immune‐mediated inflammatory diseases.

Although these findings should be interpreted cautiously given the exploratory nature of the study, they support the concept that IL‐23 may be preferentially associated with a cutaneous phenotype characterized by heliotrope rash within the spectrum of inflammatory myopathies.

## 5. Conclusion

In this study, higher serum IL‐23 levels were associated with cutaneous involvement, particularly with heliotrope rash.

## Funding

Dr. Monica Vazquez‐Del‐Mercado received funding from the Secretaría de Ciencia, Humanidades, Tecnología e Innovación (SECIHTI) through Ciencia de Frontera 2023, grant number CF‐2023‐G‐1396, and from the Universidad de Guadalajara through the Fondo Institucional Participable (FIP): Desarrollo de la Investigación y el Posgrado, Programa de Fortalecimiento de Institutos, Centros y Laboratorios de Investigación.

Dr. Andrea Aguilar‐Vazquez received funding from the SECIHTI through Estancias Posdoctorales por México 2023 and from the Universidad de Guadalajara through the Programa de Apoyo a la Mejora en las Condiciones de Producción de los Miembros del SNII (PROSNII).

## Conflicts of Interest

The authors declare no conflicts of interest.

## Supporting Information

Additional supporting information can be found online in the Supporting Information section.

## Supporting information


**Supporting Information** IL‐23 STROBE.

## Data Availability

Data sharing is not applicable to this article as no datasets were generated or analyzed during the current study.
